# Longitudinal analysis of cost and dental utilization patterns for older adults in outpatient and long-term care settings in Minnesota

**DOI:** 10.1371/journal.pone.0232898

**Published:** 2020-05-14

**Authors:** Barbara J. Smith, Michael Helgeson, Brenda Prosa, Tracy L. Finlayson, Mario Orozco, Padideh Asgari, Ian Pierce, Gregory Norman, Eliah Aronoff-Spencer

**Affiliations:** 1 Apple Tree Dental, Mounds View, Minnesota, United States of America; 2 School of Public Health, San Diego State University, San Diego, California, United States of America; 3 West Health Institute, San Diego, California, United States of America; 4 University of California San Diego, San Diego, California, United States of America; University of Utah, UNITED STATES

## Abstract

**Background:**

Dental utilization patterns and costs of providing comprehensive oral healthcare for older adults in different settings have not been examined.

**Methods:**

Retrospective longitudinal cohort data from Apple Tree Dental (ATD) were analyzed (N = 1,159 total; 503 outpatients, 656 long-term care residents) to describe oral health status at presentation, service utilization patterns, and care costs. Generalized estimating equation (GEE) repeated measures analysis identified significant contributors to service cost over the three-year study period.

**Results:**

Cohort mean age was 74 years (range = 55–104); the outpatient (OP) group was younger compared to the long-term care (LTC) group. Half (56%) had Medicaid, 22% had other insurance, and 22% self-paid. Most (72%) had functional dentitions (20+ teeth), 15% had impaired dentitions (9–19 teeth), 6% had severe tooth loss (1–8 teeth), and 7% were edentulous (OP = 2%, LTC = 11%). More in the OP group had functional dentition (83% vs. 63% LTC). The number of appointments declined from 5.0 in Year 1 (OP = 5.7, LTC = 4.4) to 3.3 in Year 3 (OP = 3.6, LTC = 3.0). The average cost to provide dental services was $1,375/year for three years (OP = $1,427, LTC = $1,336), and costs declined each year, from an average of $1,959 (OP = $2,068, LTC = $1,876) in Year 1 to $1,016 (OP = $989, LTC = $1,037) by Year 3. Those with functional dentition at presentation were significantly less costly than those with 1–19 teeth, while edentulous patients demonstrated the lowest cost and utilization. Year in treatment, insurance type, dentition type, and problem-focused first exam were significantly associated with year-over-year cost change in both OP and LTC patients.

**Conclusion:**

Costs for providing comprehensive dental care in OP and LTC settings were similar, modest, and declined over time. Dentate patients with functional dentition and edentulous patients were less costly to treat. LTC patients had lower utilization than OP patients. Care patterns shifted over time to increased preventive care and decreased restorative care visits.

## Background

Older adults and seniors are a large and growing population group in the United States (U.S.); they are retaining more teeth today than in previous generations. Nationwide, 11% of community-dwelling adults age 50 and older were edentulous, based on 2009–2014 estimates from the National Health and Nutrition Examination Survey (NHANES) [1]. Among dentate 65 and 74 year olds, 20% have untreated dental caries [2], and 60% of adults 65 years and older have periodontal disease [3]. Older adults and seniors bear a high oral disease burden [4], yet many live on limited incomes, lack dental coverage, and must pay largely out-of-pocket for dental care [5].

Cost affects utilization, and the high cost of dental care is the primary reason cited by patients for not visiting the dentist [6–8]. Dental benefits are excluded from Medicare, the primary health insurance coverage for older adults in the U.S. over age 65 [5, 9,10]. In 2015, only 38% of older adults had any dental coverage, of whom the majority (28%) had private insurance and 10% were covered through Medicaid [11]. Adult dental coverage for low-income individuals via Medicaid is an optional benefit. Three states (Alabama, Maryland, and Tennessee) do not offer dental benefits (as of 2019). In states that do offer adult dental coverage, the benefit scope varies, and may include emergency services only, or offer very limited procedures [6,12]. Minnesota’s Medicaid program provides a limited adult benefit set that includes basic diagnostic, preventive, restorative, surgical and denture services, but does not provide coverage for periodontal scaling and other services. Evidence from the 2006 and 2008 waves of the Health and Retirement Survey (HRS) showed that adults over 50 with lower income and poorer oral health do not access dental services, resulting in worse outcomes and a need for more expensive care later [13–15]. Dental utilization among seniors age 65 and older was 43.6% overall in 2014, which reflects an increase since the Affordable Care Act in 2010 [16].

There have been no recent analyses that explore costs of providing comprehensive dental care for older adults and seniors in different settings over time, including both outpatients (OP) and those in long-term care (LTC) facilities. Notably, existing available data from NHANES, HRS, and Medical Expenditure Panel Survey (MEPS) samples only include non-institutionalized individuals. The estimated 1.5 million older adults living in institutional facilities like nursing homes are missed in these national health studies [17] leaving a gap in understanding the costs for dental services in LTC settings. Older adults have varying levels of dependence that affect their ability to tend to their oral healthcare needs, which presents challenges to providing their dental care [18]. In a recent systematic review, the most common barriers reported to providing oral healthcare for dependent older adults were transportation and suitable facilities [19]. There are unique care considerations and costs associated with delivering dental care in LTC settings that have not been fully explored.

In the state of Minnesota, the Basic Screening Survey (BSS) for Older Adults conducted in 2016 focused on LTC populations and concluded that oral disease burden was high; 41% of older adults living in nursing homes had untreated caries, 43% required dental treatment, 66% had partial tooth loss (fewer than 20 teeth), and 25% were edentulous [20]. Among community-dwelling older adults in the state, 10% had lost all their teeth, and 28% had lost six or more teeth due to tooth decay or gum disease [21]. Apple Tree Dental (ATD; https://www.appletreedental.org/) is a non-profit group dental practice founded in 1985 [22,23]. Initially addressing the unmet dental needs of individuals living in LTC settings in Minnesota, ATD now serves people of all ages and abilities.

Since the start of ATD in 1985, standard billing information and diagnostic codes have been recorded in its customized information systems. The result is a unique longitudinal database that includes records for over 152,000 patients, including more than 46,000 LTC facility residents and 11,000 outpatient seniors. From this database, a cohort of older adults in both OP and LTC settings was selected and analyzed to understand oral health status, care patterns, and significant contributors to service cost for providing comprehensive dental care to new patients (separately for OP and LTC patients).

The purpose of this retrospective longitudinal cohort analysis study is to describe the oral health status, dental service utilization patterns, and cost of care for new patients in OP and LTC settings over a three-year study period. The primary outcome of interest was submitted charges (referred to as costs henceforth) for dental services at the procedure level.

## Methods

### Data sources

Data were recorded using ATD’s Open Dental Software ™ (at the time of data exportation, version 17.3. 2018. Salem, OR), an electronic health record certified by The Office of the National Coordinator for Health Information Technology (ONC). Data were reduced to a limited data set, with all other Patient Health Identifiers (PHI) removed in compliance with the Health Insurance Portability and Accountability Act of 1996 (HIPAA) safe harbor requirements (https://www.hhs.gov/hipaa/for-professionals/privacy/special-topics/de-identification/index.html).

### Study population

ATD provides comprehensive dental care, and operates two distinct care delivery models: traditional outpatient (OP) and mobile dentistry. Outpatient services for community-dwelling individuals were delivered at six regional dental centers, while mobile dentistry teams delivered on-site services to over 130 long-term care (LTC) facilities across the state of Minnesota, 90 of which were captured in this study [22,23].

**Fig 1:**
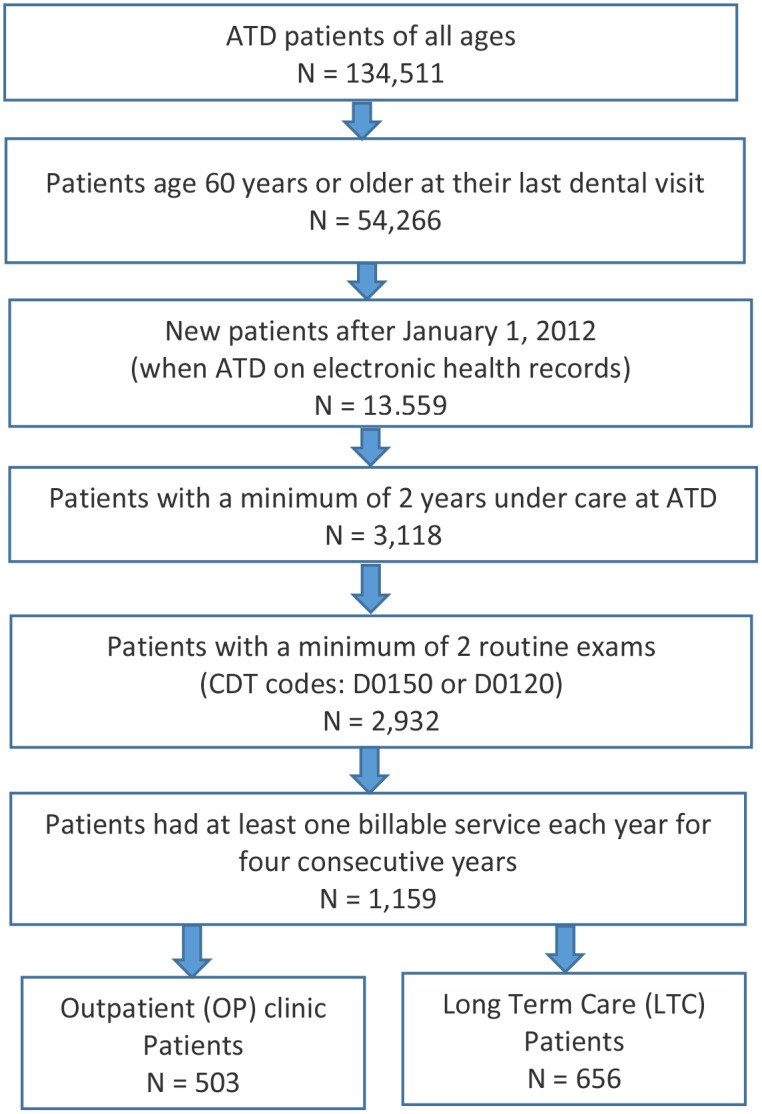
Cohort selection from Apple Tree Dental (ATD) patients.

The cohort selected for this analysis was comprised of older adults and seniors who met the following inclusion criteria: (1) new patients to ATD during 2012–2013, (2) had at least one billable service each year for four consecutive years between 2012 and 2017, (3) had at least two routine exams (American Dental Association Current Dental Terminology (ADA CDT) codes D0150 or D0120) and (4) age 60 or older at their last dental visit. The inclusion criteria captured 1,159 older adults; 503 served by the OP care delivery model and 656 in LTC served by mobile dentistry. During the course of the study, 10 patients who were initially served in the OP setting transitioned to LTC and were assigned to the LTC group. A flowchart depicts the cohort selection process (Fig 1).

The final analytic dataset included variables for cost, insurance type, demographic characteristics, care setting, procedures including dates of service, dentition type, oral health status, and designation of special treatment needs.

### Study design and institutional review board approval

This was a retrospective cohort study. Institutional review board (IRB) approval was sought for this secondary data analysis of routinely-collected, retrospective, de-identified dataset, and exemption was granted by Western IRB (reference number 1-1055253-1). No further ethics approval was required. A data sharing agreement was established between the Gary and Mary West Health Institute and Apple Tree Dental before any data were shared.

### Outcome variable of interest

The April 2015 metropolitan fee schedule for services in Twin Cities, Minnesota, was applied to all cost estimates in this analysis. Cost data was limited to billable American Dental Association Current Dental Terminology (ADA CDT) codes. To more completely capture costs, procedures that were referred to providers outside ATD were assigned a cost based on ATD fees.

There are two important points to note about costs as reported in this study. First, the reported costs are “submitted charges,” not the reimbursements actually paid by Medicaid, other dental insurance plans, or by private pay patients. During 2015, for example, Apple Tree’s reimbursements were only 45% of “costs” for Medicaid, 72% for commercial dental insurance, and 86% for private pay. During that year, Apple Tree’s total reimbursements were 51%, or about half of the submitted charges or “costs” during the year. Second, the seniors selected for this sample were seen multiple times each year over a multi-year period, and as a result had dramatically higher access to dental care than typical seniors, particularly low-income seniors. In fact, during 2015, only 43.6% of all U.S. seniors utilized dental services [16]. For these reasons, caution must be used when comparing costs as reported in this study with per-capita cost statistics, which typically include seniors who did not utilize dental services during the year.

### Primary predictor variables

Patient characteristics included age (continuous, and grouped 55–64, 65–74, 75–84, and 85+), sex (male or female), race (White, African American, Asian, other, missing which were collapsed into dichotomy of White or Other for modeling), ethnicity (Hispanic, not Hispanic, or missing), and insurance status (public/Medicaid, other insurance, or self-pay). Insurance status was identified at first and last visit. The “other” insurance category included either commercial coverage or Veterans Administration (VA). The care setting was either OP or LTC, and another variable was created to reflect the care setting’s location (urbanicity) as either urban or rural.

Procedure categories were defined by the ADA CDT categories of service and combined into five groups for analysis: Diagnostic and Preventive; Restorative; Prosthodontics-Removable; Oral Surgery; Other Services (which included Endodontics, Periodontics, fixed Prosthodontics and Adjunctive Services). Adjunctive services included anesthesia as well as house/extended care visits incurred in the LTC care model. Patients were assigned to one of four dentition types based on their tooth count at presentation: Functional dentition was defined as ≥ 20 teeth, impaired dentition (9–19 teeth), severe tooth loss (1–8 teeth), and edentulous (0 teeth) [24,25]. The presence and type of upper or lower removable prosthesis at presentation was noted (full, partial, or none). The type of first exam was categorized as either problem-focused or routine. A problem-focused exam was indicated by ADA CDT code D0140 or a documented concern that initiated the new patient appointment.

The assessment of the periodontal status at the initial visit varied; comprehensive periodontal probing was completed when possible, but in some cases, clinicians were not able to probe at all because the patient couldn’t cooperate. An American Academy of Periodontology (AAP) classification [26] may be assigned during the initial examination, at a subsequent hygiene visit or, in some instances, derived exclusively from charting notes (in the absence of a specified AAP designation). However, because of the variability in data collection, the term periodontal status is used in place of AAP classification. The use of the 1–4 categorization is an approximation and does not conform to the rigorous assignment strategy employed by the AAP classification. Rather, categories 1 or 2 reflect a picture of periodontal status that is considered healthy or reversible (gingivitis), respectively. Categories 3 and 4 are used to describe non-reversible changes, moderate or severe bone loss respectively. As in the AAP classification, categorization is done using pocket depth, bone loss, mobility, and related factors.

Three types of special treatment needs were also captured for all patients. The first category was “extra time to treat,” which reflected the need for more time to provide dental services, such as patients treated in wheelchairs, and those requiring conscious sedation and behavior management to facilitate dental care. The second category “patient unable to,” captures physical limitations such as inability to transfer into a dental chair or inability to tolerate periodontal charting. The third category was “documented findings of daily oral care,” which is a clinical determination of the patient’s ability to perform daily oral care independently, supervised, dependent on a caregiver, or if this status was unknown.

### Analytic approach

Descriptive statistics were tabulated for the entire sample, by care setting (OP and LTC), and by dentition type to characterize the cohort’s sociodemographic characteristics and oral health status at first visit. Dental utilization was reported per year by measures of central tendency for cost, as well as by number of visits, extractions, restorations, and removable prostheses delivered. Average annual cost of care for each of the three years were calculated and reported by age category, care setting (OP or LTC), dentition type, and procedure category. Chi-square tests were conducted to compare differences in demographic characteristics between the OP and LTC groups, with statistical significance set at p<0.05. Two-sample t-tests with unequal variances compared the annual costs each year, with Bonferroni corrections applied, thus the statistical significance level was set at p< (0.05/n) or p<0.01.

Generalized Estimating Equation models were used in a repeated measures analysis separately for OP and LTC to investigate the factors that contributed to variation in year-over-year cost in each group. The models include year in treatment (time indicator), age category, sex, race, insurance type, urbanicity, dentition type, problem-focused first exam, needing extra time to treat, and daily oral care status. “Patient unable to” status was excluded from the models due to high collinearity with the “extra time to treat” variable. The model outcome of cost per year was normalized via log transformation to account for skewness. Fitness between variance structures was assessed by the Akaike Information Criterion (AIC) [27].

Analyses were performed using Python (Python v2.7, Python Software Foundation, (https://www.python.org/) and SAS (SAS Enterprise Guide v7.15, 2018, SAS Software Institute, Cary, NC).

## Results

### Cohort description

**Fig 2:**
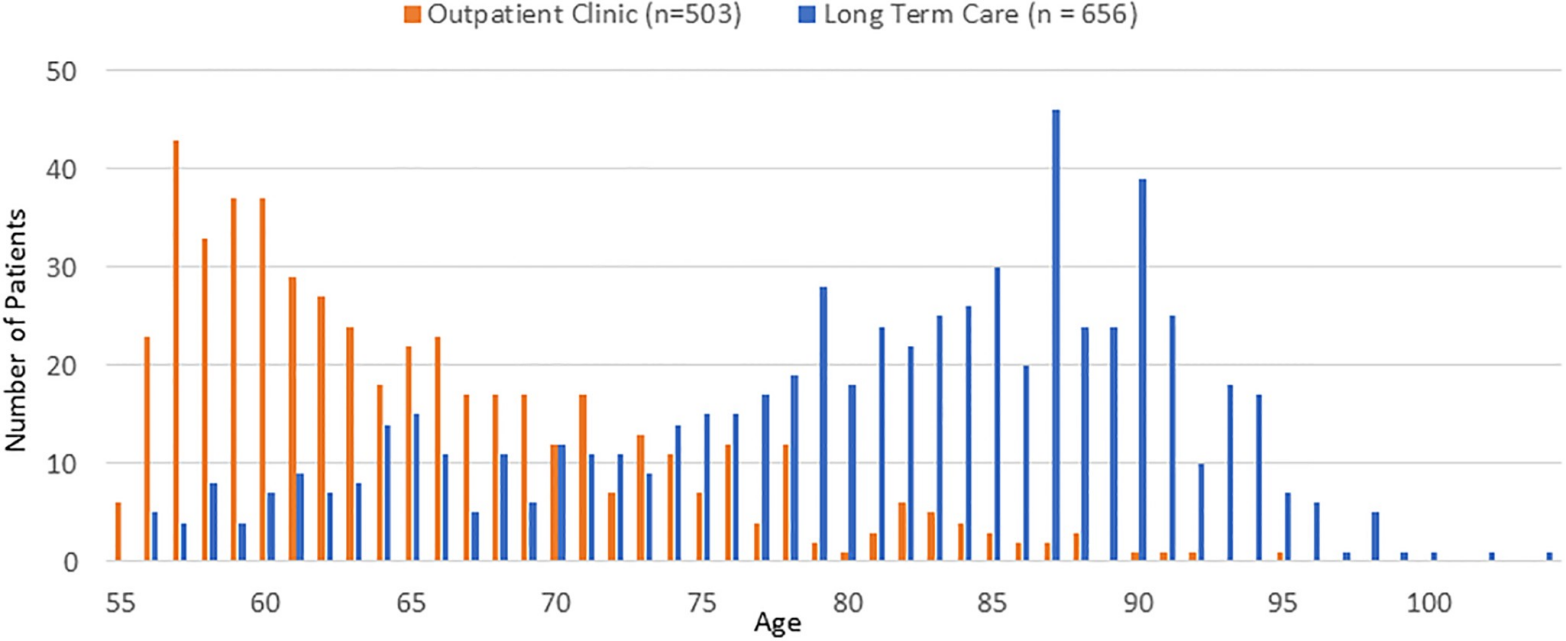
Age distribution for outpatient clinic and long-term care.

The average patient age was 74 years old (SD = 11.9, range = 55–104), but the age distributions in OP and LTC settings were mirror images (Fig 2). While 86% of the OP patients were 74 years or younger, 74% of the LTC patients were 75 years or older (chi-square(3) = 452, p<0.0001). Two-thirds of the patients were female, with 60% females in the OP setting compared to 70% females in the LTC setting. Most patients in both settings were White (91% in OP, and 90% in LTC) and Non-Hispanic (95% in OP, and 93% in LTC) (Table 1).

**Table 1:** Minnesota cohort demographics.

	Total			Outpatient			Long Term Care		
	All	Dentate	Edentulous	All	Dentate	Edentulous	All	Dentate	Edentulous
	n = 1159	n = 1077	n = 82	n = 503	n = 494	n = 9	n = 656	n = 583	n = 73
	N (%)	n (%)	n (%)	n (%)	n (%)	n (%)	n (%)	n (%)	n (%)
Average Age (St. Dev)	74.0 (11.9)	74.0 (12.0)	78.0 (10.8)	65.0 (8.0)	65.0 (8.0)	67.0 (8.8)	80.0 (10.2)	81.0 (10.1)	79.0 (10.3)
Age, Range	55–104	55–104	57–98	55–95	55–95	57–84	56–104	56–104	58–98
Age Category									
55–64	343 (30)	329 (31)	14 (17)	277 (55)	272 (55)	5 (56)	66 (10)	57 (10)	9 (12)
65–74	261 (23)	244 (23)	17 (21)	156 (31)	154 (31)	2 (22)	105 (16)	90 (15)	15 (21)
75–84	265 (23)	238 (22)	27 (33)	56 (11)	54 (11)	2 (22)	209 (32)	184 (32)	25 (34)
85+	290 (25)	266 (25)	24 (29)	14 (3)	14 (3)	0 (0)	276 (42)	252 (43)	24 (33)
Female	761 (66)	701 (65)	60 (73)	304 (60)	299 (61)	5 (56)	457 (70)	402 (69)	55 (75)
Urbanicity									
Urban	812 (70)	744 (69)	68 (83)	257 (51)	249 (50)	8 (89)	555 (85)	495 (85)	60 (82)
Rural	347 (30)	333 (31)	14 (17)	246 (49)	245 (50)	1 (11)	101 (15)	88 (15)	13 (18)
Race									
White	1049 (91)	981 (91)	68 (83)	460 (91)	453 (92)	7 (78)	589 (90)	528 (91)	61 (84)
African American	29 (3)	24 (2)	5 (6)	9 (2)	8 (2)	1 (11)	20 (3)	16 (3)	4 (5)
Asian	22 (2)	18 (2)	4 (5)	14 (3)	13 (3)	1 (11)	8 (1)	5 (1)	3 (4)
Other	5 (0)	5 (0)	0 (0)	3 (1)	3 (1)	0 (0)	2 (0)	2 (0)	0 (0)
Missing/Declined	54 (5)	49 (5)	5 (6)	17 (3)	17 (3)	0 (0)	37 (6)	32 (5)	5 (7)
Ethnicity									
Hispanic	6 (1)	6 (1)	0 (0)	4 (1)	4 (1)	0 (0)	2 (0)	2 (0)	0 (0)
Non-Hispanic	1087 (94)	1010 (94)	77 (94)	478 (95)	469 (95)	9 (100)	609 (93)	541 (93)	68 (93)
Missing/Declined	66 (6)	61 (6)	5 (6)	21 (4)	21 (4)	0 (0)	45 (7)	40 (7)	5 (7)
First Visit Payer Type									
Medicaid	654 (56)	586 (54)	68 (83)	297 (59)	288 (58)	9 (100)	357 (54)	298 (51)	59 (81)
*Other Insurance	251 (22)	245 (23)	6 (7)	112 (22)	112 (23)	0 (0)	139 (21)	133 (23)	6 (8)
Self-Pay	254 (22)	246 (23)	8 (10)	94 (19)	94 (19)	0 (0)	160 (24)	152 (26)	8 (11)
Last Visit Payer Type									
Medicaid	731 (63)	656 (61)	75 (91)	289 (57)	280 (57)	9 (100)	442 (67)	376 (64)	66 (90)
*Other Insurance	238 (21)	234 (22)	4 (5)	125 (25)	125 (25)	0 (0)	113 (17)	109 (19)	4 (5)
Self-Pay	190 (16)	187 (17)	3(4)	89 (18)	89 (18)	0 (0)	101 (15)	98 (17)	3 (4)

*Other Insurance includes Commercial and VA.

At first visit, most patients had public insurance through Medicaid (OP = 59%, LTC = 54%), followed by other insurance (OP = 22%, LTC = 21%), and self-pay (OP = 19%, LTC = 24%). By the last visit, the percentage of Medicaid patients changed; OP declined to 57%, while LTC increased to 67%.

Table 2 summarizes patient oral health status at presentation. Overall, OP patients had better oral health status. Functional dentition was observed more often among OP patients (OP = 83%; LTC = 63%). Conversely, edentulism was higher among LTC patients (OP = 2%; LTC = 11%). A full description of dentition type by age category is shown in an additional file (see S1, Dentition by Age Group and Setting). Fewer OP patients had partial or full removable prostheses than LTC patients (OP = 15% vs. LTC = 25% for upper, and OP = 9% vs. LTC = 17% for lower prostheses). At first visit, OP patients were more likely to have a problem-focused exam, compared to the LTC group (OP = 27%; LTC = 17%). A periodontal assessment status of 3 or 4 (reflecting moderate to severe bone loss) was less prevalent among the OP group compared to LTC (OP = 30%; LTC = 40%). Treatment accommodations were needed more frequently among the LTC patients. About a quarter of LTC patients needed extra time for treatment (OP = 4%; LTC = 27%) and had mobility/functional issues (OP = 4%; LTC = 26%). Many more LTC patients required supervision or were dependent on others for their daily oral care (OP 8%; LTC 43%).

**Table 2:** Oral health status, Minnesota cohort.

	Total			Outpatient			Long Term Care		
	All (N = 1159)	Dentate (n = 1077)	Edentulous (n = 82)	All (n = 503)	Dentate (n = 494)	Edentulous (n = 9)	All (n = 656)	Dentate (n = 583)	Edentulous (n = 73)
	N (%)	n (%)	n (%)	n (%)	n (%)	n (%)	n %	n %	n %
**Dentition Type**									
Functional (20–32 teeth)	829 (72)	829 (77)	0 (0)	415 (83)	415 (84)	0 (0)	414 (63)	414 (71)	0 (0)
Impaired (9–19 teeth)	174 (15)	174 (16)	0 (0)	58 (12)	58 (12)	0 (0)	116 (18)	116 (20)	0 (0)
Severe Tooth Loss (1–8 teeth)	74 (6)	74 (7)	0 (0)	21 (4)	21 (4)	0 (0)	53 (8)	53 (9)	0 (0)
Edentulous (0 teeth)	82 (7)	0 (0)	82 (100)	9 (2)	0 (0)	9 (100)	73 (11)	0 (0)	73 (100)
**Removable Prosthesis—Upper Present at First Visit**									
None	919 (79)	897 (83)	22 (27)	429 (85)	427 (86)	2 (22)	490 (75)	470 (81)	20 (27)
*Full	145 (13)	85 (8)	60 (73)	33 (7)	26 (5)	7 (78)	112 (17)	59 (10)	53 (73)
Partial	95 (8)	95 (9)	0 (0)	41 (8)	41 (8)	0 (0)	54 (8)	54 (9)	0 (0)
**Removable Prosthesis—Lower Present at First Visit**									
None	1003 (87)	975 (91)	28 (34)	460 (91)	458 (93)	2 (22)	543 (83)	517 (89)	26 (36)
*Full	62 (5)	8 (1)	54 (66)	11 (2)	4 (1)	7 (78)	51 (8)	4 (1)	47 (64)
Partial	94 (8)	94 (9)	0 (0)	32 (6)	32 (6)	0 (0)	62 (9)	62 (11)	0 (0)
**First Exam**									
Routine Exam	913 (79)	848 (79)	65 (79)	366 (73)	358 (72)	8 (89)	547 (83)	490 (84)	57 (78)
Problem-Focused Exam	246 (21)	229 (21)	17 (21)	137 (27)	136 (28)	1 (11)	109 (17)	93 (16)	16 (22)
**Periodontal Assessment									
1	-	229 (21)	-	-	119 (24)	-	-	110 (19)	-
2	-	460 (43)	-	-	228 (46)	-	-	232 (40)	-
3	-	335 (31)	-	-	122 (25)	-	-	213 (37)	-
4	-	43 (4)	-	-	23 (5)	-	-	20 (3)	-
Unknown	-	10 (1)	-	-	2 (0)	-	-	8 (1)	-
***Extra Time to Treat									
None	962 (83)	892 (83)	70 (85)	482 (96)	474 (96)	8 (89)	480 (73)	418 (72)	62 (85)
Any	197 (17)	185 (17)	12 (15)	21 (4)	20 (4)	1 (11)	176 (27)	165 (28)	11 (15)
****Patient Unable To									
None	968 (84)	900 (84)	68 (83)	485 (96)	478 (97)	7 (78)	483 (74)	422 (72)	61 (84)
Any	191 (16)	177 (16)	14 (17)	18 (4)	16 (3)	2 (22)	173 (26)	161 (28)	12 (16)
**Daily Oral Care Status**									
Independent	753 (65)	715 (66)	38 (46)	423 (84)	418 (85)	5 (56)	330 (50)	297 (51)	33 (45)
Supervised	186 (16)	173 (16)	13 (16)	19 (4)	18 (4)	1 (11)	167 (25)	155 (27)	12 (16)
Dependent	133 (11)	125 (12)	8 (10)	18 (4)	17 (3)	1 (11)	115 (18)	108 (19)	7 (10)
Unknown	87 (8)	64 (6)	23 (28)	43 (9)	41 (8)	2 (22)	44 (7)	23 (4)	21 (29)

Dentition is determined by tooth count at presentation. Routine Exam includes comprehensive exam and in rare cases recall exam due to Medicaid restrictions. Problem Focused Exam includes limited exam and custom codes for Tooth Concern and Denture Concern. Periodontal disease index determined by chart review.

*Dentate patients with full upper or lower dentures had at least one tooth in the opposing arch.

**Periodontal Disease Index reported for the dentate population only (n = 1077); edentulous excluded.

*** Treated in Wheelchair is the most common Extra Time status (n = 163, 14%), followed by Frequent Stops and Starts (n = 44, 4%) Gentle Hand and Head Holding (n = 15, 1%), and Behavioral Management (n = 10, 1%).

**** Unable to Transfer to Dental Chair is the most common Patient Unable To status (n = 151, 13%), followed by Unable to Communicate Needs (n = 48, 4%) and Unable to Tolerate Dental Radiographs (n = 36, 3%).

***, **** Patients can have 0, 1, or any combination of Extra Time and Patient Unable statuses.

### Cost and visits by year, care setting, and dentition type

**Fig 3:**
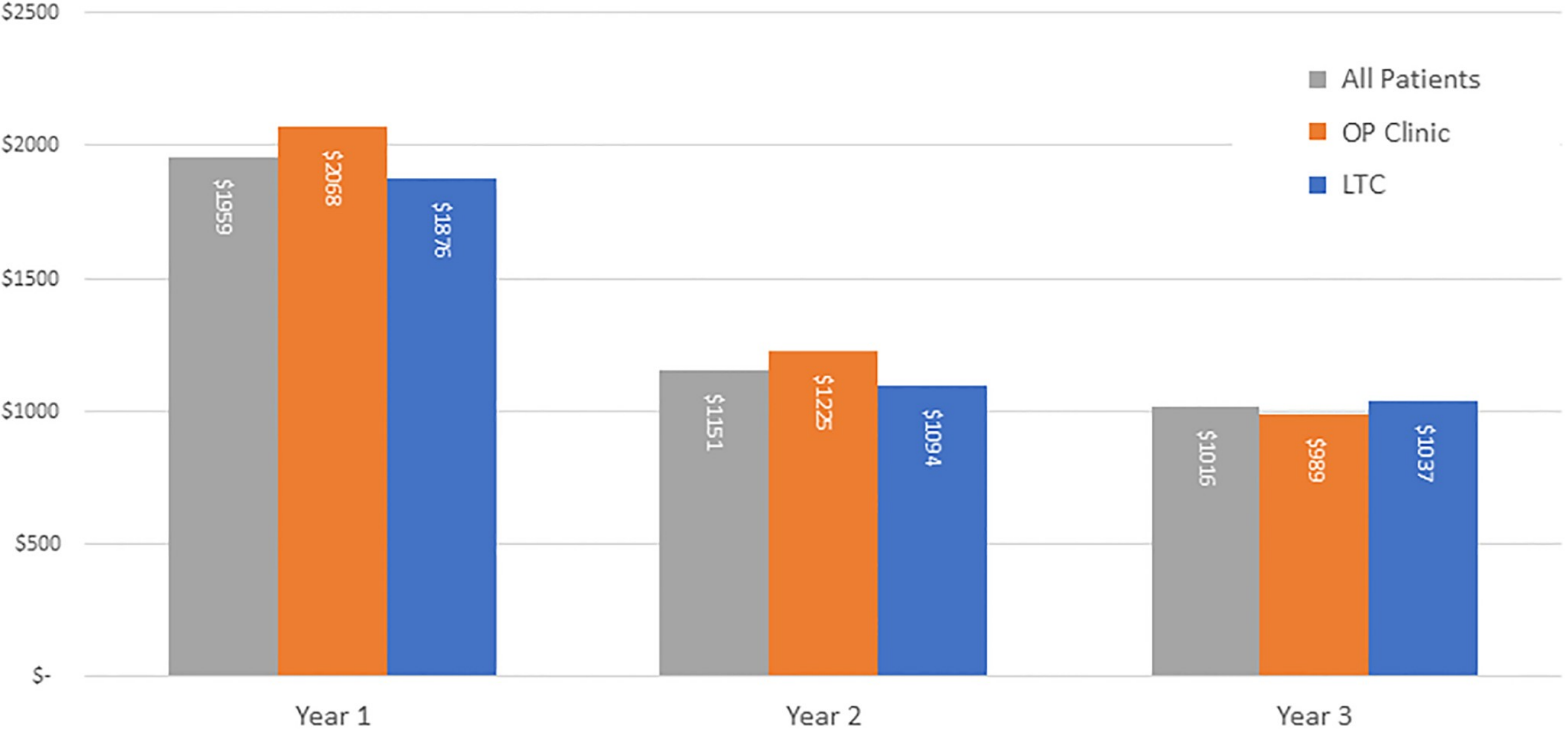
Average cost per year, by care setting.

Average cost was highest in the first year for both care settings and decreased with each subsequent year. For OP patients, the average cost decreased from $2,068 in Year 1 to $989 in Year 3. These OP cost decreases were statistically significant between Year 1 and Year 2 (t-test statistic = 7.43, p<0.000), and from Year 2 to Year 3 (t-test statistic = 2.78, p = 0.006). For LTC patients, the average cost decreased from $1,876 in Year 1 to $1,037 in Year 3 (Fig 3). These LTC decreases were statistically significant between Year 1 and Year 2 (t-test statistic = 8.56, p<0.000), but not from Year 2 to Year 3 (t-test statistic = 0.85, p = 0.396). The decline overall for all patients from Year 1 to Year 2 was statistically significant (t-test statistic = 11.32, p<0.000).

Mirroring cost, the average number of visits was highest in Year 1 and decreased in each subsequent year (Table 3). For OP patients, the average number of visits declined from 5.7 in Year 1 to 3.6 by Year 3. For LTC patients, the average number of visits declined from 4.4 in Year 1 to 3.0 by Year 3. Dentate patients averaged more appointments per year than those who were edentulous. Patients in the impaired and severe tooth loss dentition groups had more visits which may reflect the multiple appointments required to treat numerous teeth and surfaces or properly fit new prostheses.

**Table 3:** Annual dental cost and visits, Minnesota cohort.

Average Cost Per Year ($)		Year 1		Year 2		Year 3		3 Year Averages	
		Mean (St.Dev)	Range	Mean (St.Dev)	Range	Mean (St.Dev)	Range	Mean (St.Dev)	Range*
Total	N = 1159	1,959 (2,017)	83–15,070	1,151 (1,356)	54–11,089	1,016 (1,199)	53–9,517	1,375 (1,052)	54–15,070
	Functional (n = 829)	1,789 (1,907)	108–15,070	1,150 (1,303)	54–11,089	1,005 (1,132)	53–9,517	1,315 (1,040)	53–15,070
	Impaired (n = 174)	2,651 (2,253)	136–10,019	1,323 (1,468)	79–8,642	1,238 (1,396)	72–8,769	1,737 (1,163)	72–10,019
	Severe Tooth Loss (n = 74)	2,433 (2,299)	83–13,816	1,301 (1,694)	54–8,009	936 (1,293)	54–5,967	1,557 (981)	54–13,816
	Edentulous (n = 82)	1,784 (1,953)	83–6,497	661 (1,192)	54–4,471	725 (1,258)	54–4,495	1,057 (747)	54–6,497
Outpatient	All (n = 503)	2,068 (2,073)	83–13,816	1,225 (1,476)	54–11,089	989 (1,213)	53–8,769	1,427 (1,071)	53–13,816
	Functional (n = 415)	1,907 (1,955)	108–12,117	1,204 (1,413)	54–11,089	984 (1,166)	53–7,708	1,365 (1,040)	53–12,117
	Impaired (n = 58)	2,913 (2,291)	136–8,658	1,611 (1,933)	79–8,642	1,215 (1,563)	107–8,769	1,913 (1,252)	79–8,769
	Severe Tooth Loss (n = 21)	2,631 (3,032)	83–13,816	855 (1,141)	54–3,914	611 (884)	54–3,847	1,366 (1,041)	54–13,816
	Edentulous (n = 9)	2,743 (1,867)	83–4,258	580 (1,249)	54–3,903	621 (1,315)	54–4,108	1,315 (478)	54–3,903
Long Term Care	All (n = 656)	1,876 (1,970)	83–15,070	1,094 (1,255)	95–8,647	1,037 (1,189)	54–9,517	1,336 (1,036)	54–15,070
	Functional (n = 414)	1,671 (1,852)	150–15,070	1,096 (1,182)	95–8,647	1,026 (1,099)	95–9,517	1,264 (1,038)	95–15,070
	Impaired (n = 116)	2,520 (2,232)	274–10,019	1,179 (1,152)	149–6,412	1,250 (1,312)	72–7,019	1,650 (1,111)	72–10,019
	Severe Tooth Loss (n = 53)	2,355 (1,965)	192–7,020	1,478 (1,848)	105–8,009	1,065 (1,409)	125–5,967	1,633 (955)	105–8,009
	Edentulous (n = 73)	1,666 (1,943)	83–6,497	671 (1,193)	125–4,471	738 (1,260)	54–4,495	1,025 (770)	54–6,497
Visits Per Year									
Total	N = 1159	5.0 (3.5)	1–23	3.5 (2.7)	1–22	3.3 (2.5)	1–20	3.9 (2.1)	1–23
	Functional (n = 829)	4.7 (3.2)	1–23	3.4 (2.5)	1–19	3.2 (2.3)	1–20	3.8 (2.0)	1–23
	Impaired (n = 174)	6.2 (3.9)	1–18	4.0 (3.3)	1–19	3.9 (3.0)	1–17	4.7 (2.5)	1–19
	Severe Tooth Loss (n = 74)	5.7 (4.0)	1–19	3.8 (3.4)	1–22	3.2 (2.9)	1–14	4.2 (2.2)	1–22
	Edentulous (n = 82)	4.5 (3.8)	1–14	2.3 (2.1)	1–9	2.4 (2.2)	1–9	3.1 (1.7)	1–14
Outpatient	All (n = 503)	5.7 (3.9)	1–23	3.9 (2.9)	1–19	3.6 (2.8)	1–20	4.4 (2.3)	1–23
	Functional (n = 415)	5.4 (3.6)	1–23	3.8 (2.7)	1–19	3.5 (2.6)	1–20	4.2 (2.2)	1–23
	Impaired (n = 58)	7.8 (4.3)	1–18	5.0 (4.1)	1–18	4.6 (3.8)	1–17	5.8 (2.8)	1–18
	Severe Tooth Loss (n = 21)	7.2 (5.3)	1–19	3.1 (2.4)	1–8	2.6 (2.1)	1–8	4.3 (2.4)	1–19
	Edentulous (n = 9)	6.3 (3.7)	1–11	2.7 (2.7)	1–9	1.8 (1.7)	1–6	3.6 (1.1)	1–11
Long Term Care	All (n = 656)	4.4 (3.0)	1–21	3.1 (2.4)	1–22	3.0 (2.2)	1–15	3.5 (1.9)	1–22
	Functional (n = 414)	4.1 (2.6)	1–21	3.0 (2.1)	1–15	2.9 (1.9)	1–15	3.3 (1.7)	1–21
	Impaired (n = 116)	5.4 (3.4)	1–16	3.5 (2.6)	1–19	3.5 (2.4)	1–12	4.1 (2.1)	1–19
	Severe Tooth Loss (n = 53)	5.1 (3.2)	1–13	4.1 (3.7)	1–22	3.4 (3.2)	1–14	4.2 (2.2)	1–22
	Edentulous (n = 73)	4.3 (3.8)	1–14	2.2 (2.0)	1–9	2.5 (2.2)	1–9	3.0 (1.7)	1–14

*These ranges represent the minimum and maximum cost and visit count across all three years within the sample

**Fig 4:**
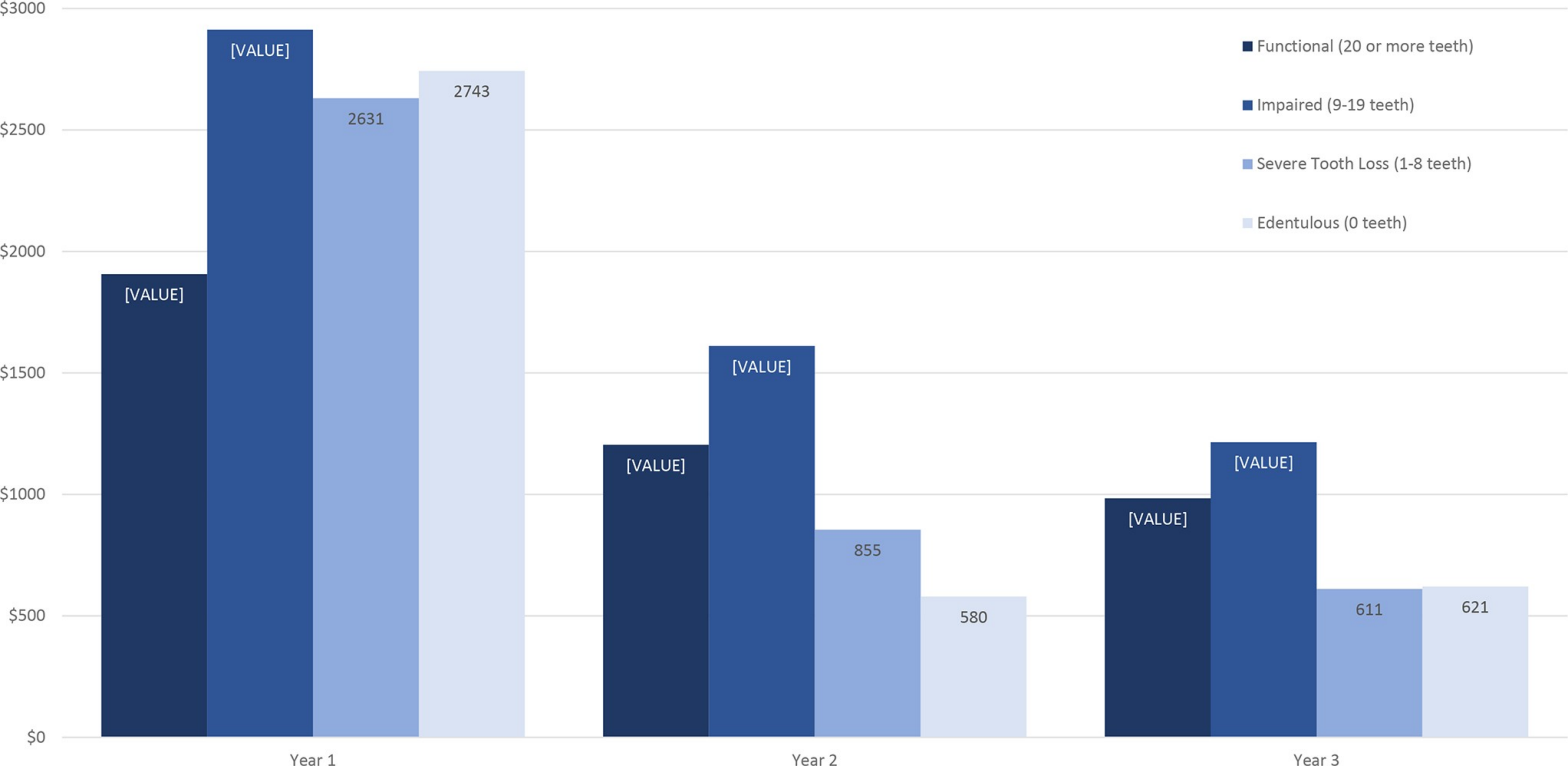
Average cost per year by dentition type, outpatient clinic.

**Fig 5:**
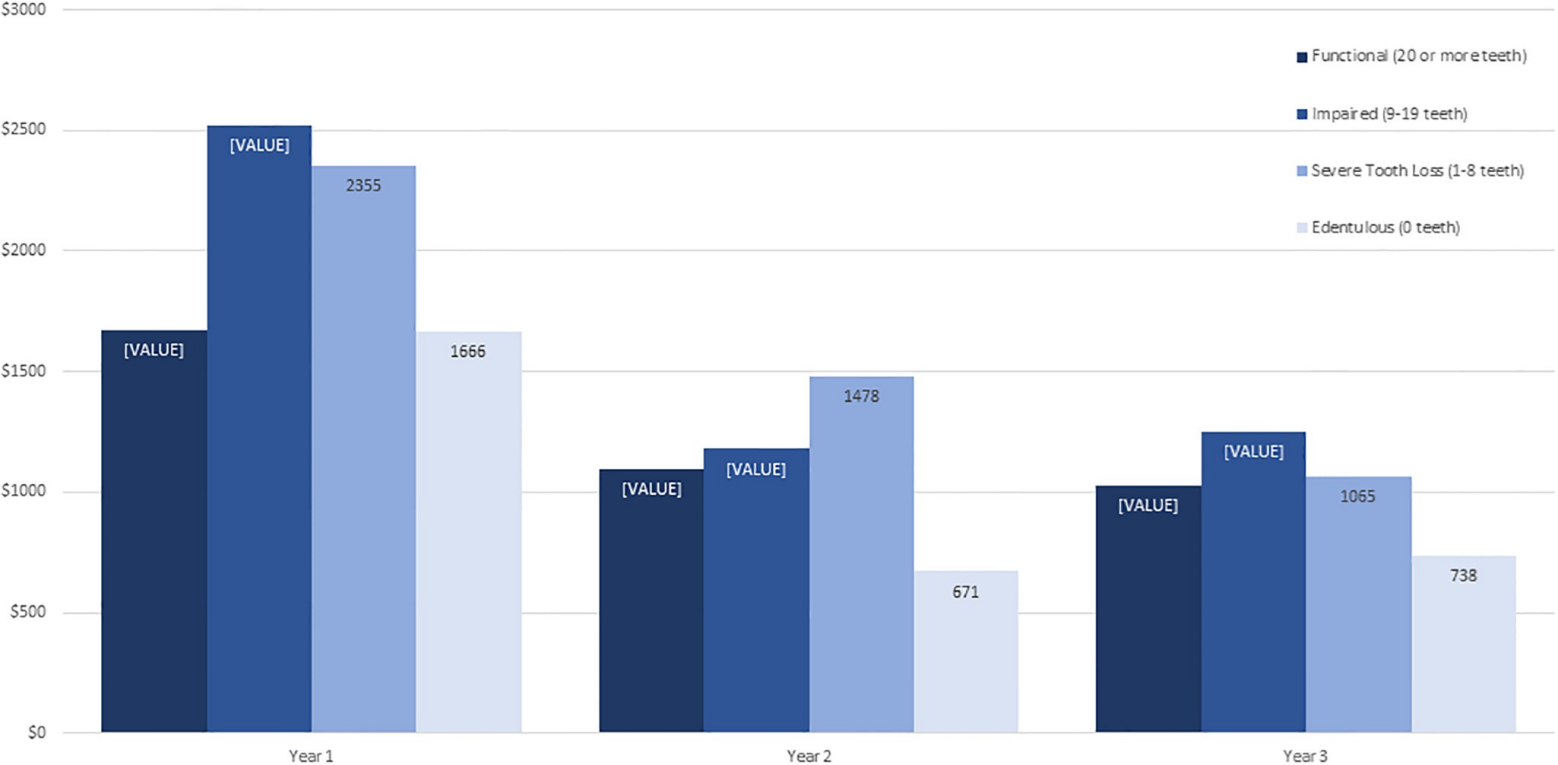
Average cost per year by dentition type, long-term care.

Overall, cost decreased across the three-year study for all dentition types in both care settings. In the OP setting (Fig 4), patients with functional dentition had the lowest cost in Year 1, relative to other dentition groups. Patients with impaired dentition had the highest costs in all three years. Despite the severe tooth loss and edentulous OP groups having high costs in Year 1, the costs dropped substantially in Years 2 and 3, where they were the least costly groups to care for. With the exception of the impaired dentition group, the three-year average costs for other dentition types were very comparable in the OP group (functional $1,365, severe tooth loss $1,366, edentulous $1,315). In the LTC setting (Fig 5), the functional dentition and edentulous groups were the least costly in each of the three years. Those with impaired dentition had the highest average Year 1 cost, at $2,520, followed by the severe tooth loss group (average Year 1 cost $2,355). The three-year average costs for these two dentition types were very comparable in the LTC group (impaired $1,650, severe tooth loss $1,633).

### Service utilization categories by year in treatment and care setting

Average annual utilization for extractions, restorations, and removable prostheses delivered were highest in Year 1 (Table 4). In Year 1, more OP patients received any oral surgery extractions compared to LTC (OP = 35%; LTC = 23%, edentulous excluded), and more restorations (OP = 70%; LTC = 47%, edentulous excluded), and removable prostheses compared to LTC (OP = 15%; LTC = 14%, including edentulous). The number of patients receiving extractions, restorations, and prostheses declined in years 2 and 3.

**Table 4:** Annual dental utilization by setting and dentition type, unique patients and services per year, Minnesota cohort.

Unique Patients and Services Per Year		Year 1			Year 2			Year 3		
		Unduplicated Patients	Total Services	Utilizer Average	Unduplicated Patients	Total Services	Utilizer Average	Unduplicated Patients	Total Services	Utilizer Average
**Extractions**										
***Total***	*N = 1159*	*325*	*1012*	*3*.*1*	*189*	*420*	*2*.*2*	*168*	*357*	*2*.*1*
***Outpatient***	*n = 503*	*175*	*558*	*3*.*2*	*85*	*167*	*2*.*0*	*78*	*138*	*1*.*8*
Functional	n = 415	142	436	3.1	75	131	1.7	68	115	1.7
Impaired	n = 58	27	103	3.8	10	36	3.6	9	22	2.4
Severe Tooth Loss	n = 21	7	19	2.7	0	0	0.0	1	1	1.0
Edentulous	n = 9	-	-	-	-	-	-	-	-	-
***Long Term Care***	*n = 656*	*149*	*454*	*3*.*0*	*104*	*253*	*2*.*4*	*90*	*219*	*2*.*4*
Functional	n = 414	93	282	3.0	73	190	2.6	63	136	2.2
Impaired	n = 116	40	126	3.2	19	34	1.8	18	55	3.1
Severe Tooth Loss	n = 53	16	46	2.9	12	29	2.4	9	28	3.1
Edentulous	n = 73	-	-	-	-	-	-	-	-	-
**Restorations**										
***Total***	*N = 1159*	*661*	*2577*	*3*.*9*	*531*	*1671*	*3*.*1*	*495*	*1497*	3.0
***Outpatient***	*n = 503*	*353*	*1419*	*4*.*0*	*271*	*878*	*3*.*2*	*251*	*735*	2.9
Functional	n = 415	304	1209	4.0	233	764	3.3	210	617	2.9
Impaired	n = 58	40	189	4.7	32	106	3.3	34	103	3.0
Severe Tooth Loss	n = 21	9	21	2.3	6	8	1.3	7	15	2.1
Edentulous	n = 9	-	-	-	-	-	-	-	-	-
***Long Term Care***	*n = 656*	*308*	*1158*	3.8	*260*	*793*	3.1	*244*	*762*	3.1
Functional	n = 414	221	817	3.7	193	576	3.0	179	588	3.3
Impaired	n = 116	67	270	4.0	52	169	3.3	54	156	2.9
Severe Tooth Loss	n = 53	20	71	3.6	15	48	3.2	11	18	1.6
Edentulous	n = 73	-	-	-	-	-	-	-	-	-
Removable Prostheses Delivered*										
***Total***	*N = 1159*	*166*	*260*	*1*.*6*	*88*	*123*	*1*.*4*	*55*	*81*	*1*.*5*
***Outpatient***	*n = 503*	*74*	*112*	*1*.*5*	*41*	*59*	*1*.*4*	*25*	*32*	*1*.*3*
Functional	n = 415	34	49	1.4	26	34	1.3	17	20	1.2
Impaired	n = 58	24	36	1.5	11	18	1.6	6	8	1.3
Severe Tooth Loss	n = 21	10	15	1.5	3	5	1.7	1	2	2.0
Edentulous	n = 9	6	12	2.0	1	2	2.0	1	2	2.0
***Long Term Care***	*n = 656*	*92*	*148*	*1*.*6*	*47*	*64*	*1*.*4*	*30*	*49*	*1*.*6*
Functional	n = 414	22	29	1.3	18	22	1.2	5	9	1.8
Impaired	n = 116	26	43	1.7	9	10	1.1	7	12	1.7
Severe Tooth Loss	n = 53	19	29	1.5	11	17	1.5	8	11	1.4
Edentulous	n = 73	25	47	1.9	9	15	1.7	10	17	1.7

*Removable prostheses include upper and lower; partial, complete, and immediate dentures.

### Cost per service category per year in treatment, OP and LTC

**Fig 6:**
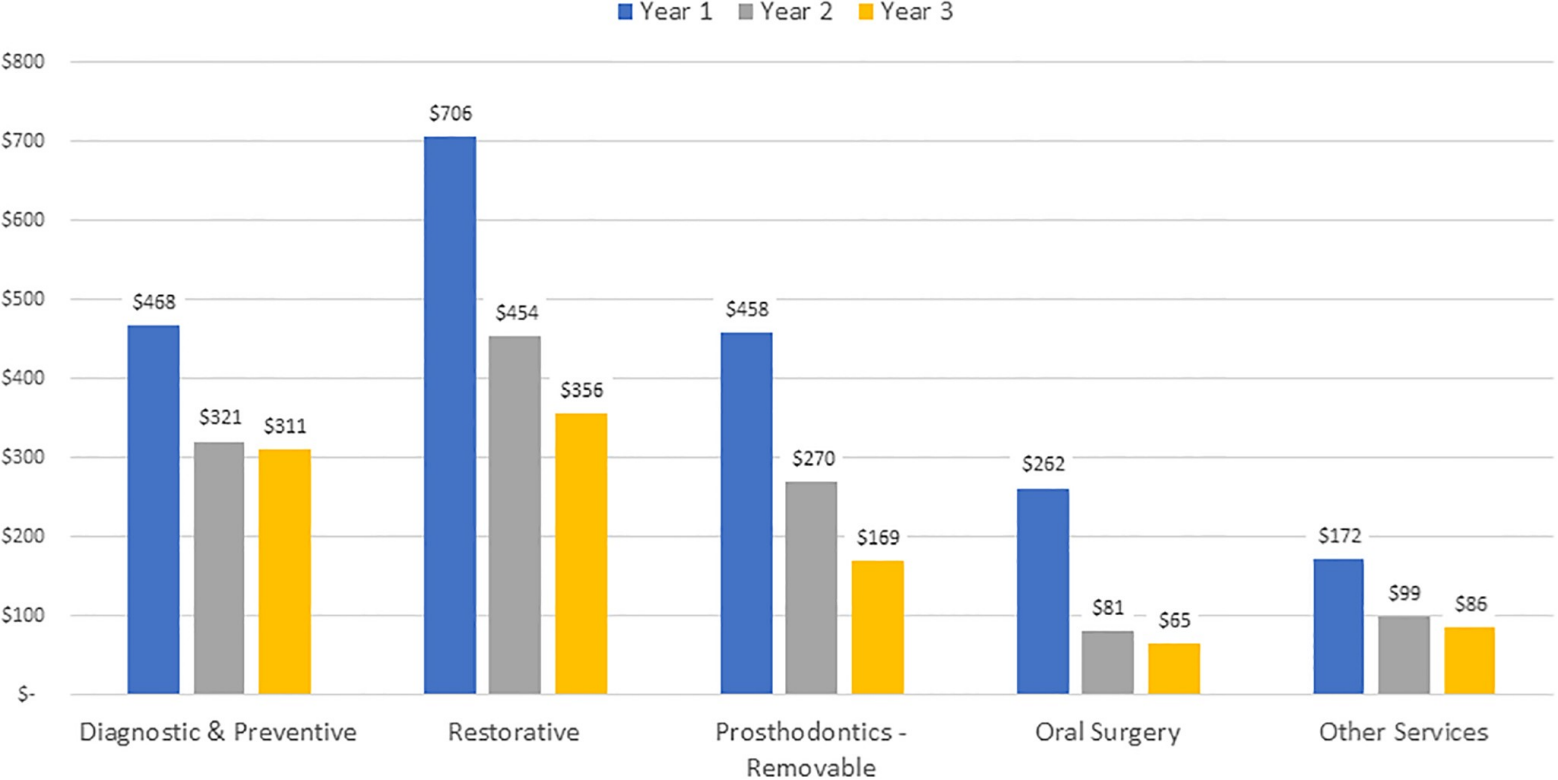
Cost by service category, outpatient clinic.

**Fig 7:**
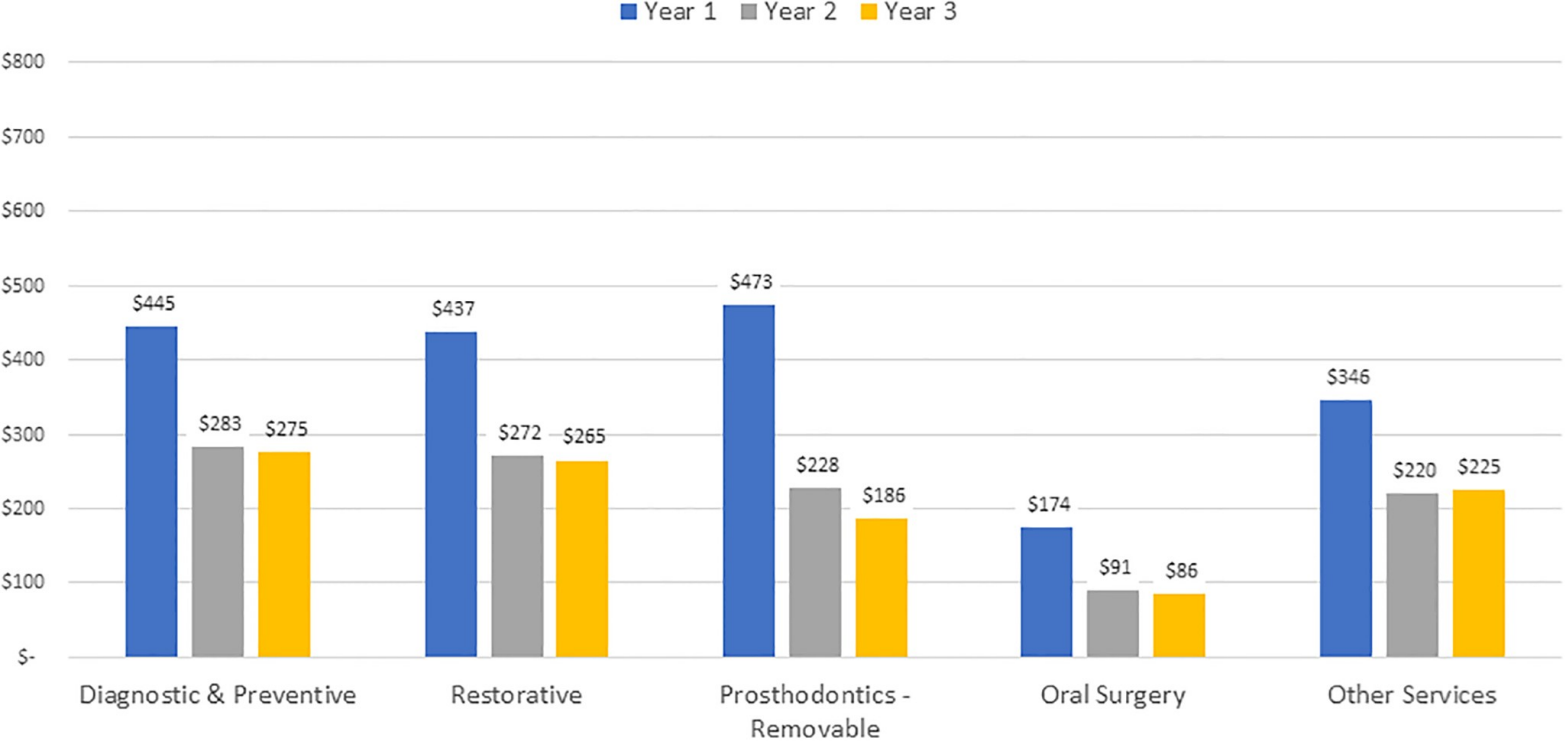
Cost by service category, long-term care.

The average annual cost by service category and care setting are presented in Figs 6 and 7 for OP and LTC groups, respectively. Overall, the average annual cost declined within each service category in Years 1 through 3 for both settings. For OP patients, restorative services represented the largest share of costs, averaging $706 in Year 1, declining to $356 in Year 3. For LTC patients, services in the removable prosthodontics category had the highest costs, averaging $473 in Year 1, declining to $186 by Year 3. The averages include repairs and adjustments as well as fabrication of new dentures. All service categories showed a similar decline in cost for both groups. The “Other Services” category incurred significantly greater cost for LTC compared to OP. This is explained by the finding that the majority (85%) of the Other Services cost for LTC was from the mobile dentistry care delivery model’s use of ADA CDT code D9410 house/extended care facility call, subsequently referred to as “Facility Visit Charge.” Boxplots of cost per year by care setting and dentition type are available as S2 and S3, respectively.

### Generalized estimating equations repeated measure analysis

Year in treatment, insurance type, dentition type, and problem-focused first exam were significantly associated with year-over-year cost change in the OP setting (Table 5). Age, race, sex, and urbanicity were not significant. Beta estimates describe year-over-year deviation from reference category average cost and are not intended to identify the features associated with total cost. For example, the OP model beta estimate of -0.4355 for severe tooth loss reflects the decrease in cost over three years relative to the functional dentition reference category, not lower total cost. Patients with functional dentition had an average cost of $1,365 for three years (refer to Table 3), which was very comparable to the average cost of $1,366 for three years among the group with severe tooth loss. The negative beta estimate reflects a decline in changes in cost for the severe tooth loss group from Year 1 to subsequent years.

**Table 5:** Generalized estimating equations repeated measure analysis in outpatient settings.

Characteristics	N	Year 1	Year 2	Year 3	Outpatient Model N = 503	
		Average cost	Average cost	Average cost	Beta Estimate	p-value
**Year in treatment**		$2068	$1225	$989	-0.4165	< .0001
**Age**						
55–64	277	$2135	$1267	$1019	-	-
65–74	156	$1918	$1177	$908	0.0287	0.6712
75–84	56	$2230	$1019	$1101	0.0049	0.9599
≥ 85	14	$1783	$1764	$835	0.1444	0.4141
**Sex**						
Male	199	$2227	$1306	$954	-	-
Female	304	$1964	$1173	$1011	-0.1014	0.0832
**Race**						
White	460	$2044	$1225	$987	-	-
Other	43	$2332	$1228	$1007	-0.0189	0.8573
**Insurance**						
Medicaid	297	$2401	$1351	$1081	-	-
Other Insurance	112	$1591	$991	$858	-0.2936	0.0001
Self-Pay	94	$1588	$1107	$852	-0.3465	< .0001
**Urbanicity**						
Urban	257	$2027	$1172	$1016	-	-
Rural	246	$2112	$1281	$959	-0.0386	0.5223
**Dentition**						
Functional	415	$1907	$1204	$984	-	-
Impaired	58	$2913	$1611	$1215	0.1887	0.0375
Severe Tooth loss	21	$2631	$855	$611	-0.4335	0.0029
Edentulous	9	$2743	$580	$621	-0.7918	0.0003
**Exam**						
Routine	366	$1805	$1126	$979	-	-
Problem-focused	137	$2773	$1491	$1014	0.2436	0.0002
**Extra Time to Treat**						
No	482	$2114	$1231	$988	-	-
Yes	21	$1032	$1102	$1011	-0.3142	0.0778
**Daily Oral Care**						
Independent	423	$2108	$1232	$1005	-	-
Supervised	19	$1939	$1221	$931	-0.1820	0.2486
Dependent	18	$1096	$1114	$1088	-0.1460	0.4345
Unknown	43	$2141	$1206	$811		

In the LTC setting model (Table 6), year in treatment, insurance type, dentition type, and problem-focused first exam were significantly associated with year-over-year cost change. Additionally, needing extra time to treat and supervised daily oral care status were also significantly associated with variation in year-over-year cost for LTC patients. Age, race, sex, and urbanicity were not significant in the LTC model.

**Table 6:** Generalized estimating equations repeated measure analysis in Long-Term Care (LTC) settings.

Characteristics	N	Year 1	Year 2	Year 3	LTC N = 656	
		Average cost	Average cost	Average cost	Beta Estimate	p-value
**Year in treatment**		$1876	$1094	$1037	-0.3200	< .0001
**Age**						
55–64	66	$2221	$1460	$897	-	-
65–74	105	$2373	$1216	$1189	0.0703	0.4038
75–84	209	$1692	$1099	$1127	0.0335	0.6656
≥ 85	276	$1743	$957	$944	-0.0308	0.6926
**Sex**						
Male	199	$2197	$1293	$1144	-	-
Female	457	$1735	$1008	$991	-0.0854	0.0757
**Race**						
White	589	$1824	$1085	$1030	-	-
Other	67	$2325	$1181	$1094	0.0066	0.9243
**Insurance**						
Medicaid	357	$2222	$1190	$1207	-	-
Other Insurance	139	$1384	$998	$910	-0.2597	< .0001
Self-Pay	160	$1529	$965	$768	-0.2601	< .0001
**Urbanicity**						
Urban	555	$1937	$1085	$1059	-	-
Rural	101	$1539	$1148	$915	0.0454	0.4425
**Dentition**						
Functional	414	$1671	$1096	$1026	-	-
Impaired	116	$2520	$1179	$1250	0.1393	0.0144
Severe Tooth loss	53	$2355	$1478	$1065	-0.1462	0.0649
Edentulous	73	$1666	$671	$738	-0.9424	< .0001
**Exam**						
Routine	547	$1698	$1088	$1004	-	-
Problem-focused	109	$2766	$1126	$1204	0.1748	0.0020
**Extra Time to Treat**						
No	480	$2029	$1117	$1087	-	-
Yes	176	$1457	$1032	$902	-0.2058	< .0001
**Daily Oral Care**						
Independent	330	$1775	$1149	$995	-	-
Supervised	167	$2209	$1193	$1175	0.1229	0.0143
Dependent	115	$1618	$956	$1056	-0.0157	0.7895
Unknown	44	$2071	$674	$777		

## Discussion

Our analysis found that costs for providing comprehensive dental care to older adults and seniors in OP and LTC settings were similar and modest, and declined overtime. Costs were highest in Year 1, then declined in each subsequent year over the three-year study period for new patients in both OP and LTC settings. Costs were modest overall, and comparable between those seeking care on an OP basis and those living in LTC facilities, though those in LTC had fewer visits. The major drivers of cost in both groups were having impaired dentition (and severe tooth loss in LTC only), having Medicaid insurance, and having a problem-focused first exam at presentation.

All patients in the cohort received dental care from ATD for the first time in the first year of the three-year study period. Overall, the cost for dental services was highest during the first year and decreased significantly thereafter. This may suggest that many new patients did not access oral health services in the previous months or years, and therefore the burden of disease at presentation may have been relatively high; however, data about prior insurance or utilization was unknown. Older patients with continuous dental care are more likely to have their needs assessed and met regularly and may lead to stabilization in the evolution of oral diseases and rehabilitation of functionality [28]. Managing oral diseases and restoring functionality may provide these patients with the benefits of appropriate mastication, speech, and self-esteem that contributes to their well-being, and help control comorbidities, polypharmacy and prevention of early mortality for this age group [29].

Dentition type at first visit was important to examine in relation to the patient’s care needs and utilization patterns and affected the subsequent cost for care. The OP cohort had a higher proportion with functional dentition, while the LTC cohort had a higher proportion of edentulism, severe tooth loss, and impaired dentition. Older adults with impaired dentition (9–19 teeth present) in both OP and LTC settings, and those with severe tooth loss (1–9 teeth) in LTC were more costly compared to those with functional dentition (20+ teeth). Edentulous patients were the least costly overall, and also showed the greatest cost decline per year of any dentition status group. This cost can likely be attributed to providing new prostheses as edentulous older adults use their new dental benefits primarily in their first year of coverage. Despite the lower overall cost, edentulism is the least desirable outcome.

Prevention and early diagnosis of oral diseases may bring costs down, as evidenced by the lower cost of care for the functional dentition group (among dentate older adults). It is important to consider that many older adults will become care-dependent as their overall physical and mental functionality deteriorate with time [17]. Furthermore, caregivers in general, including informal and often untrained caregivers for community-dwelling older adults, prioritize other daily activities and competing appointments for other medical needs over oral care, potentially resulting in poor oral health outcomes [30]. Older adults themselves may not prioritize oral healthcare, especially when faced with mobility limitations and other health concerns [31–33]. Oral hygiene is often poor for nursing home residents, and the needs are considerable and often unmet [34–37]. In several facilities, care is limited to emergencies and tooth retention is not a priority because funding is not available, despite the Federal mandate that nursing facilities provide access to dental services [38,39]. Further research could attempt to determine the cost associated with dentition as number of teeth decrease from the functional level of 20 and the number of pairs in functional occlusion.

A key finding was that a problem-focused exam on first visit was significantly associated with increased costs. Patients in the OP group were more likely to have had a problem-focused first exam, suggesting there was a pent-up backlog of needs to treat. These patients may need to rely on others to help them identify the need for dental care, scheduling an appointment, and transportation. While this study did not directly explore barriers to accessing care, other studies of community-dwelling older adults have documented high levels of unmet oral health needs, especially among homebound elderly [33, 40,41]. Navigating access challenges may be more difficult for older adults living independently, and for those with increased functional dependency. The lower rate of problem-focused first exams in the LTC population may be attributed to the ATD care model, which brings dental care to the LTC facility, thus overcoming access challenges experienced by community-dwelling older adults.

Another factor that affected costs was the need for extra time for treatment. Almost all patients identified as needing extra time were seen in the LTC setting, and the most common reasons for needing extra time were: treated in wheelchair, frequent stops and starts, gentle hand and head holding, and behavioral management. Needing more time for care was associated with significantly reduced cost, likely reflecting the fact that fewer procedures could be accomplished during appointments. Another reason for reduced costs could be that more complex and costly procedures were not suitable for these patients. LTC patients had fewer visits overall, and more time is required to provide the needed care for this subgroup. Identifying patients that require extra time to treat facilitates appropriate treatment planning, scheduling and care delivery.

Older adults use dental services often, especially those with higher incomes and wealth [9,42]. Care patterns may look very different for higher income older adults than those with public coverage. In this cohort, the number of visits by older adults in both groups decreased year by year, and costs for the various procedure categories from diagnostic and preventive to oral surgery decreased uniformly throughout the three-year study period as well. Among the OP group, restorative procedures, diagnostic and preventive activities, and removable prostheses represented the largest proportion of the cost.

For those receiving care in nursing facilities, costs were equally distributed between preventive and diagnostic procedures and restorations, followed by removable prostheses. Care delivered in nursing homes required ATD to mobilize equipment and personnel to these facilities, thus 20% of the fees billed for this cohort was allocated to the deployment of equipment and personnel at each facility. This is a special code (CDT Code D9410) which allows a professional visit once per individual per day to patients living in nursing homes, long-term care facilities, and hospice settings. Only 17% of patients required additional time to receive dental care, however, this is an important aspect of resource allocation and utilization to consider, especially in nursing homes. There are practical implications for delivering care in LTC settings. Different oral healthcare delivery models, supported by an LTC interprofessional team that includes a logistics coordinator, are needed to bring care on-site using portable equipment [23,43].

The three-year average cost incurred in this study for both OP and LTC settings was $1,375, which is a modest average of submitted charges for this unique sample of seniors who gained access to dental care consistently over a multi-year period. MEPS is a national dataset that includes cost information for health services, including dental care. The MEPS 2015 cost estimate for dental care for older adults over age 64 living in communities was $913. However, MEPS does not include institutionalized individuals in their study sample. The 2016 Medicare program payment per traditional Medicare enrollee for Part B was about $5,000 [44]. Reimbursements to Apple Tree in 2015 from all payers averaged only 51% of submitted charges, so the three-year average cost of $1,375 translates into actual public and private reimbursements of only $701. Given that the costs for treating this unique sample of seniors with very high dental utilization rates is relatively low, the expected costs for a new Medicare Part B dental benefit would be significantly less than $701. Despite the relatively low cost, dental care is seldom covered for older adults in Medicare, and out-of-pocket expenses are disproportionately high for dental services overall, and for older adults specifically [11].

This retrospective longitudinal analysis contributes to the literature by characterizing the cost for providing comprehensive dental care for older adults and seniors. There is a paucity of data with detailed records on the utilization of dental services in LTC settings. This hinders an already fragmented healthcare system from describing utilization and estimating the cost and benefit of dental services. As a result, stakeholders lack information regarding the impact that dental coverage could have on the overall health. This unique longitudinal study quantifies the cost of providing comprehensive dental care and utilization of services over a three-year period.

This analysis has important implications for payers and policymakers considering providing comprehensive dental coverage for older adults and seniors. Policymakers need to address the oral health needs of older adults and consider adding coverage for comprehensive oral health benefits. There is a large number of older adults and seniors in the U.S. that are living longer and retaining more teeth than in previous generations, putting them at higher risk to develop dental and other oral health-related diseases. Oral diseases are preventable and readily treated, however, older adults of all socioeconomic levels forgo visits to the dentist because they consider these services costly [45]. The evidence in this study will help consumers, policymakers, and payers understand the needs of seniors and the cost to offer a dental benefit.

This study provides evidence about cost and utilization patterns for a cohort of older adults and seniors in Minnesota residing in both community and LTC settings. The three-year study period allowed tracking of changes in annual costs over time. The LTC data makes a unique contribution to the literature. The limitations of this study include the following: This sample was homogenous in terms of race (White) and ethnicity (non-Hispanic) and exhibited lower rates of edentulism than national averages. This cohort appeared representative of older adults in the state of Minnesota in terms of race/ethnicity but are not reflective of the racial/ethnic diversity across the nation, and results may not be generalizable [46]. Roughly 60% of claims in this study were incurred by Medicaid beneficiaries, which is significantly higher compared to national averages of 10% for Americans over 64 with public dental coverage [11]. The patient mix reflects the mission of ATD to serve those patients eligible for public programs. Patients in this study had at least one billable dental service each year; thus, the cost of caring for patients who had infrequent episodic care patterns was not captured, and many older adults do not obtain dental care annually. In 2015, only 47% of Americans over 64 had a dental visit [11].

Additional studies with other samples are needed to analyze cost and dental utilization patterns for older adults living in varied settings. Longer-term follow-up may illuminate practical and logistic considerations related to the changing health needs of aging adults, particularly among those transitioning from living in the community to LTC facilities. Future studies should also explore how dentition type, different treatments, and delivery settings affect dental outcomes and overall health status to show oral care services are an integral part of the overall cost of care for older adults and seniors.

## Conclusions

Costs for providing comprehensive dental care to older adults and seniors in OP and LTC settings were modest and declined over time. Patients with functional dentition and edentulous patients were least costly to treat. Care patterns shifted over time to increased preventive care and decreased restorative care visits and prostheses. LTC patients overall had lower utilization than OP patients.

## Supporting information

S1 Fig Dentition by age group and setting. (PPTX)

S2 Fig Cost year 1–3 for OP and LTC. (PPTX)

S3 Fig Cost in year 1–3 by dentition type. (PPTX)
