# Supplementary material for: Longitudinal analysis of cost and dental utilization patterns for older adults in outpatient and long-term care settings in Minnesota
Source: PLoS One. 2020 May 14;15(5):e0232898. doi: 10.1371/journal.pone.0232898 (PMC7224465; doi:10.1371/journal.pone.0232898)
Supplement: S1 Fig — (PPTX) [file pone.0232898.s001.pptx]

## Slide 1
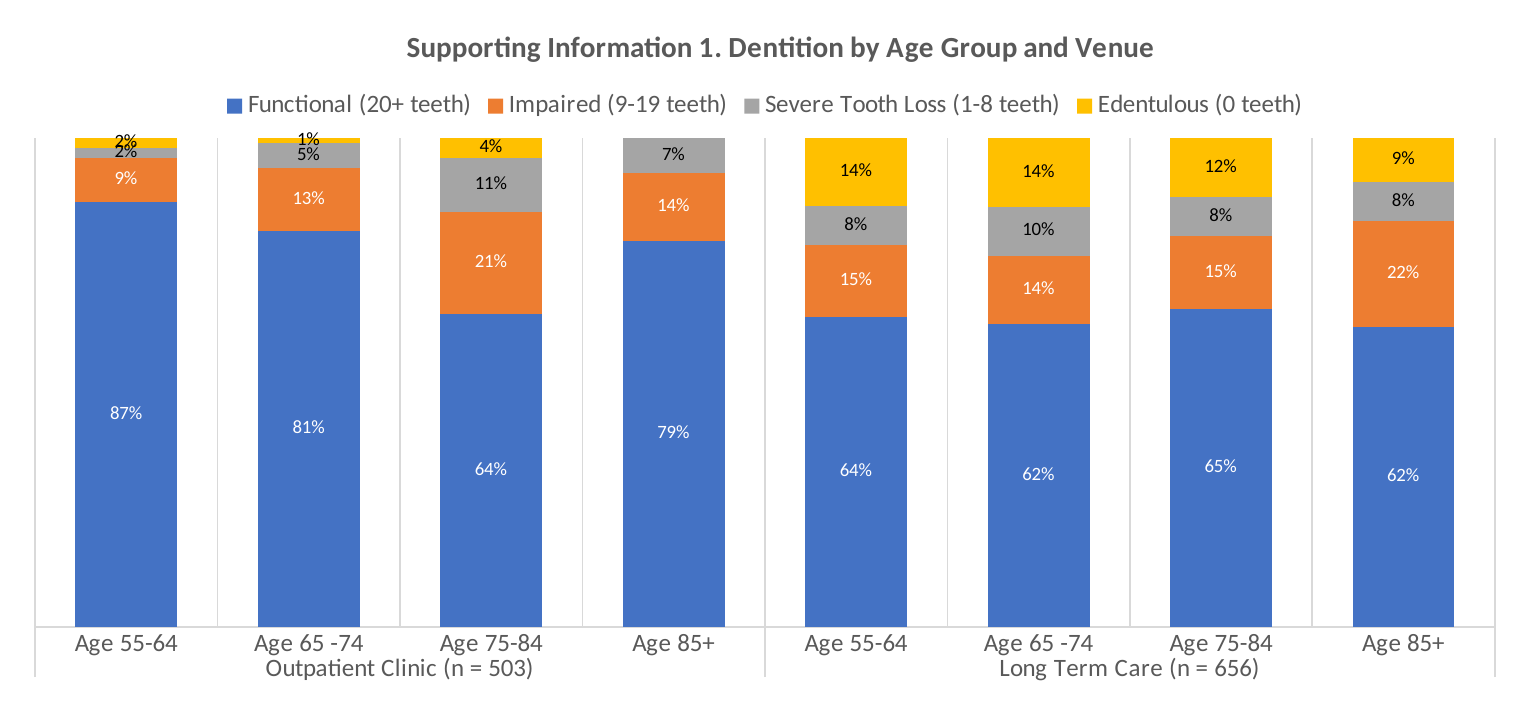

### Chart: Supporting Information 1. Dentition by Age Group and Venue
| Category | Functional (20+ teeth) | Impaired (9-19 teeth) | Severe Tooth Loss (1-8 teeth) | Edentulous (0 teeth) |
|---|---|---|---|---|
| Age 55-64 | 0.87 | 0.09 | 0.02 | 0.02 |
| Age 65 -74 | 0.81 | 0.13 | 0.05 | 0.01 |
| Age 75-84 | 0.64 | 0.21 | 0.11 | 0.04 |
| Age 85+ | 0.79 | 0.14 | 0.07 | None |
| Age 55-64 | 0.64 | 0.15 | 0.08 | 0.14 |
| Age 65 -74 | 0.62 | 0.14 | 0.1 | 0.14 |
| Age 75-84 | 0.65 | 0.15 | 0.08 | 0.12 |
| Age 85+ | 0.62 | 0.22 | 0.08 | 0.09 |
