# Supplementary figures and images for: Longitudinal analysis of cost and dental utilization patterns for older adults in outpatient and long-term care settings in Minnesota
Source: PLoS One. 2020 May 14;15(5):e0232898. doi: 10.1371/journal.pone.0232898 (PMC7224465; doi:10.1371/journal.pone.0232898)

## Slide 1
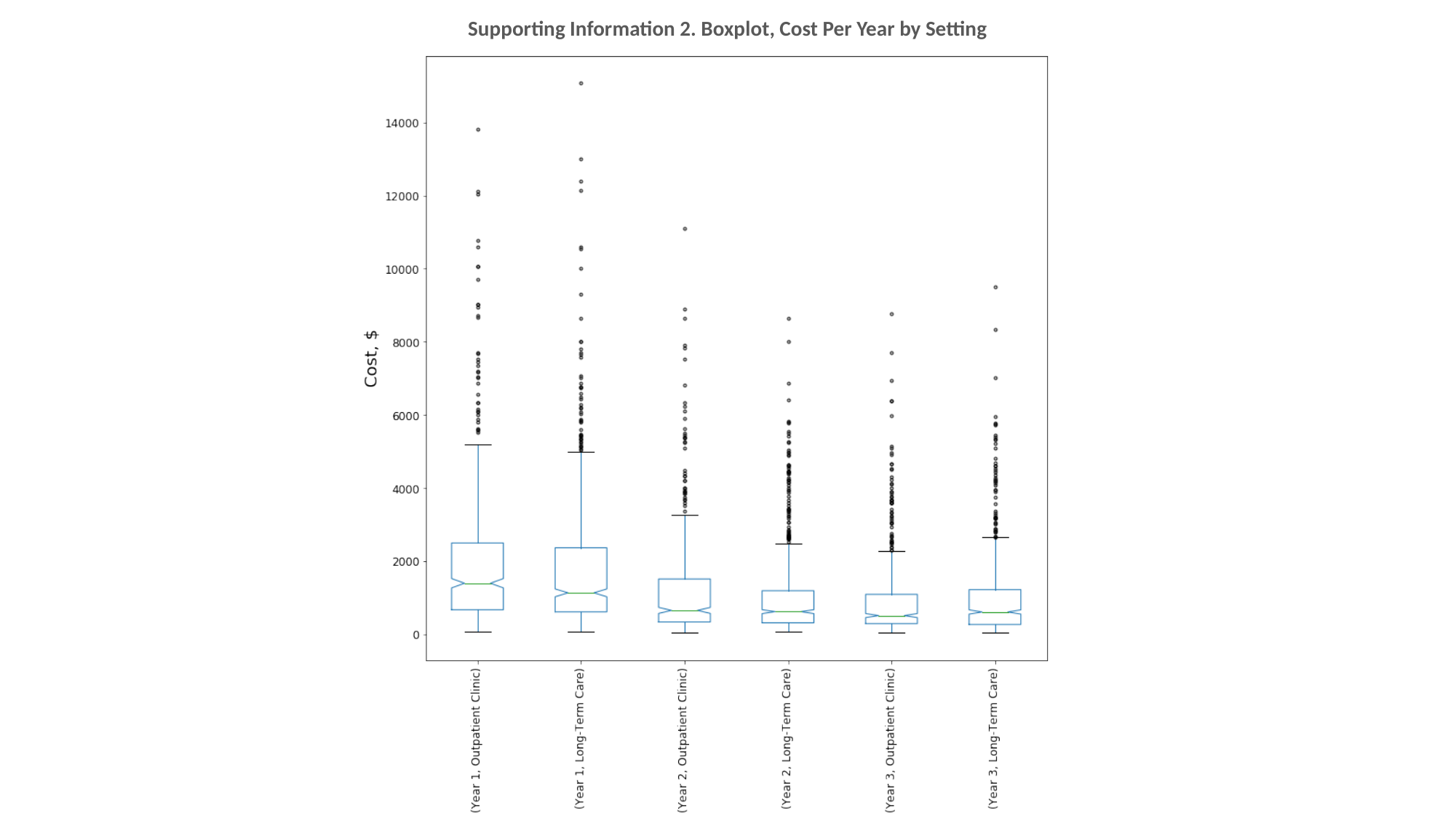

Supporting Information 2. Boxplot, Cost Per Year by Setting

Supplement: S2 Fig — (PPTX) [file pone.0232898.s002.pptx]

## Slide 1
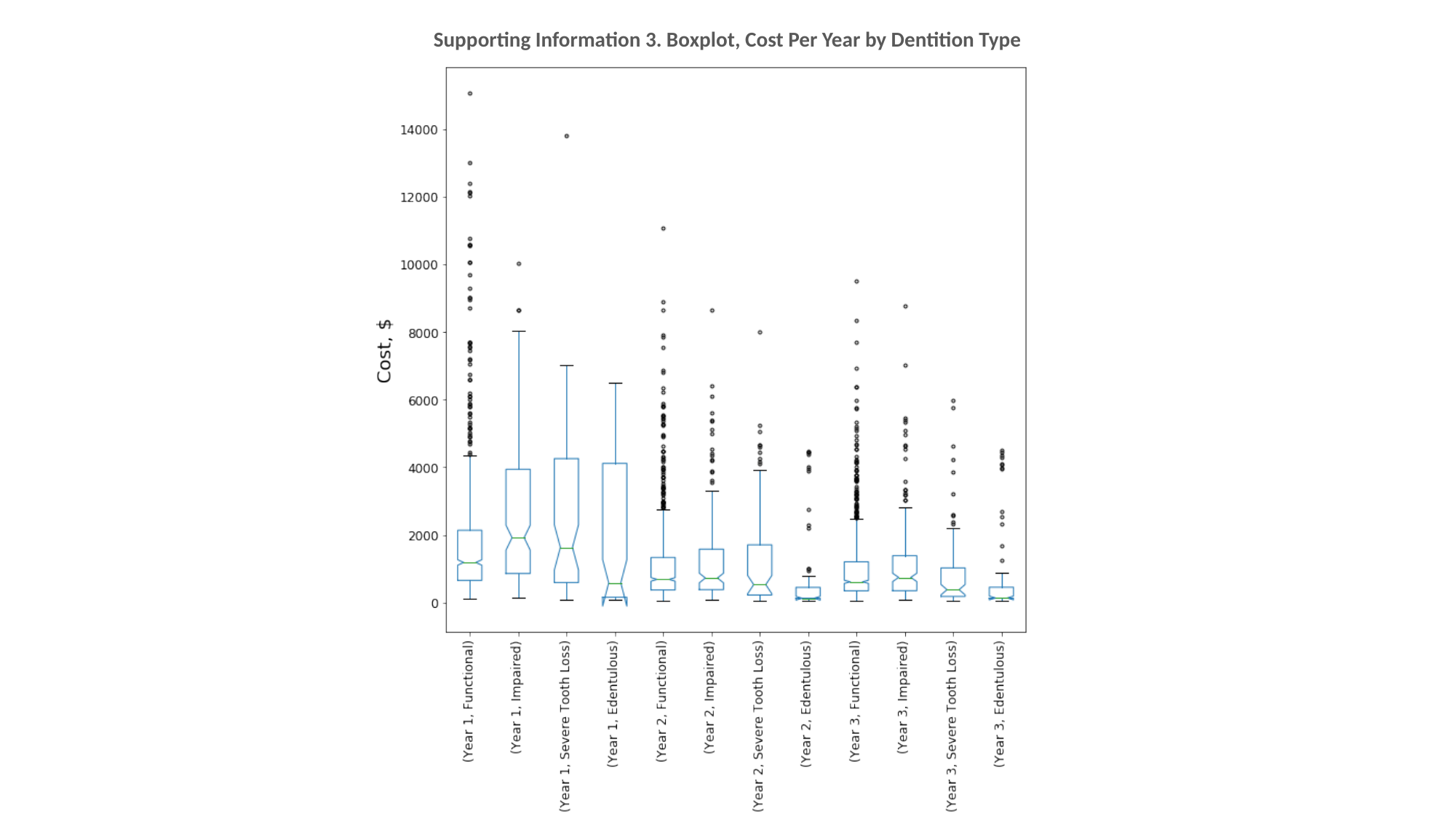

Supporting Information 3. Boxplot, Cost Per Year by Dentition Type

Supplement: S3 Fig — (PPTX) [file pone.0232898.s003.pptx]
